# Genome‐wide association studies of multiple sclerosis

**DOI:** 10.1002/cti2.1018

**Published:** 2018-05-31

**Authors:** Chris Cotsapas, Mitja Mitrovic

**Affiliations:** ^1^ Departments of Neurology and Genetics Yale School of Medicine New Haven CT USA; ^2^ Broad Institute of MIT and Harvard Cambridge MA USA

**Keywords:** disease risk genetics, genome‐wide association studies, immune‐mediated disease, multiple sclerosis

## Abstract

Large‐scale genetic studies of multiple sclerosis have identified over 230 risk effects across the human genome, making it a prototypical common disease with complex genetic architecture. Here, after a brief historical background on the discovery and definition of the disease, we summarise the last fifteen years of genetic discoveries and map out the challenges that remain to translate these findings into an aetiological framework and actionable clinical understanding.

## Introduction

Multiple sclerosis [MS, (MIM 126200)] is a neurological disorder of the central nervous system (CNS), resulting from an autoimmune attack on CNS white matter. The disease course often results in progressively decreasing motor function and is the most frequent cause of neurological disability in young adults. Over two million people worldwide suffer from MS, with over 75% of these being women. After the description of the disease by Charcot in 1868, MS was gradually recognised as a distinct, multifaceted clinical entity.^1^ The discovery of contrast agents for microscopy in the early 20th century catalysed the description of MS lesion pathology as a result of inflammation and myelin damage around blood vessels in the brain.^2^ In this golden age of bacteriology, it was assumed that the causes of MS were extrinsic, and the field searched for infectious causes to no avail.

The eventual discovery that immune cells caused myelin destruction in a primate model resembling MS^3^ finally put the field on the right track in the 1930s, and the discovery of an immunoglobulin signature in MS patient cerebrospinal fluid^4^ – still in use as a diagnostic tool today – firmly cemented the idea that MS is an autoimmune disease. Meanwhile, as medical practice became more advanced after the Second World War, patients were increasingly seen by neurologists specialising in MS, who began to compile long‐term cohorts of patients.^5^ It rapidly became obvious that the disease is geographically segregated^6^ and aggregates in certain families and that siblings and offspring of people with MS are far more likely to develop the disease themselves.^7^

## Early genetic studies

This realisation that the disease was genetic prompted the search for pathogenic genes, but it took diligent work for two decades to finally discover the first genetic risk factors for MS: three serological alleles of the human leucocyte antigens (HLA), encoded in the major histocompatibility complex^8^, ^9^, ^10^, ^11^ (MHC, chromosome 6p21). As the molecular biology of the immune system was unveiled, it was natural to ask whether these were also involved in MS pathogenesis. Candidate gene studies in cohorts of tens or hundreds of individuals at the loci encoding T‐cell receptor alpha^12^, ^13^ and beta^14^, ^15^ loci, the immunoglobulin heavy‐chain genes^16^, ^17^ and the gene for myelin basic protein,^18^, ^19^ among others, produced inconsistent findings.^20^, ^21^ As became obvious in retrospect, such studies are underpowered to detect risk alleles for common complex disease, suffer from population stratification and other artefacts and often assess genes that have broad relevance to the immune system but do not drive disease risk *per se*.^22^

The development of genetic maps covering much of the genome led to linkage analyses in extended MS affected families from a number of countries, primarily of European ancestry.^23^, ^24^, ^25^, ^26^, ^27^, ^28^, ^29^, ^30^, ^31^, ^32^ These validated the HLA association but showed no significant linkage to loci outside the MHC. Recognising that the small sample size of these studies limited power to detect non‐MHC linkages, the genetic analysis of multiple sclerosis in Europeans (GAMES) consortium was created to perform a genome‐wide association screen across multiple populations using microsatellites and pooled DNA.^23^ Although extraordinary as a collaboration for the time, this effort also failed to find non‐MHC loci. The linkage era culminated in a further collaborative effort by The International Multiple Sclerosis Genetics Consortium (IMSGC) also formed to pool resources and samples to conduct well‐powered studies. The IMSGC typed 4506 single nucleotide polymorphisms (SNPs) in 730 multiplex families and again found no significant linkage peaks outside the MHC, although a handful of suggestive signals were present.^24^ Although largely negative, these studies strongly supported the notion that MS is not caused by a small amount of mutations of large effect, but is likely to be due to many small risk effects spread across the genome.

## Genome‐wide association studies

The completion of the human genome sequencing project led to the development of complete catalogues of common genetic variation across the genome, and concomitant technologies to assay these variants in a cost‐effective and high‐throughput manner.^25^, ^26^ This technological development enabled the profiling of thousands of samples in a single study and prompted a shift away from family studies, where samples are necessarily limited and ascertainment challenging, to population‐based association studies comparing unrelated cases and controls.^27^ These genome‐wide association studies (GWAS) compare allele frequency at each variant position of the genome between cases and controls, with significant differences implying an association to disease. The often‐inconsistent results of candidate gene studies and the biases that drove them led to the adoption of robust statistical thresholds for significance in GWAS and a standard of requiring replication in independent samples.^22^ The currently acceptable standard is a significance level of *P *<* *5 × 10^−8^, which is equivalent to *P *<* *0.05 after Bonferroni correction for the number of independent tests in the genome given linkage disequilibrium between common variants.^28^ These studies have demonstrated that the common disease–common variant hypothesis of human diseases^29^ is broadly true, where disease risk is driven by many common variants, each of which explains a small fraction of the risk in a population.

In 2007, the first GWAS in MS looked at 1540 parent‐affected offspring trios and identified two loci outside the MHC, encoding the interleukin‐2 receptor (*IL‐2RA*) and the interleukin‐7 receptor (*IL‐7RA*), respectively.^30^ Several other loci showed some evidence of association, but fell short of strict genome‐wide significance thresholds; these have been subsequently validated in larger studies. The three significant findings were simultaneously replicated in independent studies from the United Kingdom, the United States and the Nordic countries.^31^, ^32^ This opened the floodgates, with several successive studies GWAS and meta‐analysis followed in rapid succession, so that by 2011, common variants in 26 genomic loci had been associated with MS risk and independently replicated, but clearly only explained a fraction of MS risk attributable to genetic factors.^33^, ^34^, ^35^, ^36^, ^37^, ^38^, ^39^, ^40^, ^41^, ^42^ These studies collectively showed that non‐MHC MS risk alleles have modest effects on disease (odds ratios < 1.2) and that even larger sample sizes (over 10 000 cases and controls) would be needed to identify more loci.^22^ A further expansion of the IMSGC resulted in a collaborative GWAS of 9772 cases and 17 376 controls, again of European descent, in 2011.^43^ This study replicated 23 of 26 previously identified associations and identified 29 novel risk loci. The number of significant associations made robust *post hoc* pathway analyses possible, and it became evident that these loci are strongly enriched for genes acting in T‐cell activation and proliferation pathways. In addition, refinement of the associations in the HLA region showed that just four variants are sufficient to account for the risk previously attributed to extended haplotype alleles spanning hundreds of kilobases (kb) and many tens of genes. A further study, this time on a targeted array (the ImmunoChip^44^) in 29 300 MS cases and 50 794 unrelated healthy individuals, identified 48 new susceptibility variants, bringing the total number of MS risk variants to 110 at 103 discrete loci outside the MHC.^45^

Most recently, the IMSGC has completed an even larger GWAS including over 115 000 cases and controls. This latest report brings the total number of MS risk associations to 233, including 200 autosomal variants outside the MHC, one on the X chromosome and 32 independent effects in the broader MHC locus, covering both classical and nonclassical gene regions.^46^ Again, careful pathway, transcriptomic and epigenetic enrichment analyses suggest T‐cell biology is a major feature of the disease, but also highlight the involvement of many other components of both adaptive and innate immunity in pathogenesis. All these effects combined explain 19.2% of the total heritability for MS. The 32 MHC effects accounted for 4% of the overall heritability, with the bulk of the remaining signal resident in the other regions of the genome associated with MS risk. However, a small portion – approximately 2% of the overall heritability – resides in regions that either did not show suggestive association in the initial GWAS or that failed to replicate in independent samples, suggesting that there remain additional loci to be found (Table 1 summarises all these findings).

**Table 1:** Genome‐wide association studies have discovered increasing numbers of MS risk loci over time. Over the last decade, increasing sample sizes have powered dramatic advances in GWAS discoveries

Year	Cases	Controls	Significance threshold	Internal replication	Replication cases	Replication controls	Significant loci after/before replication^a^	References
2007	931	2431	1 × 10^−4^	1 step	2322	2987	2/1	30
2008	242	242	0.01 (pooling based)	2 steps	100; 275	100; 275	2/2	34
2009	978	883	1 × 10^−4^	Selected SNPs	974	883	1/5	36
2009	1618	3413	1 × 10^−4^	1 step	2256	2310	2/6	37
2009	2624	7220	1 × 10^−3^	1 step	2215	2116	10/6	35
2010	68	136	1 × 10^−3^	2 steps	711; 3859	1029; 9110	8/7	38
2010	882	872	5 × 10^−8^	1 step	1775	2005	11/10	42
2010	592	825	10 × 10^−6^	No	NA	NA	3	39
2011	5545	12 153	5 × 10^−8^	Meta‐analysis	NA	NA	3	40
2011	9772	17 376	1 × 10^−4.5^	1 step	4218	7296	52/102	43
2013	14 498	24 091	1 × 10^−4^	1 step	14 802	26 703	103/135	45
2017	14 802	26 703	1 × 10^−5^	2 steps	20 282; 12 267	18 956; 22 625	200/207	46

a Locus count excludes the extended MHC.

## The role of the MHC

The first MS risk associations discovered were three serological alleles of the HLA.^8^, ^9^, ^10^, ^11^ Since then, a great deal of effort has been expended to better characterise these associations both genetically and functionally, although we still do not understand how changes to antigen display increase risk for MS or any other autoimmune disease.^47^ One of the main challenges to interrogating the MHC is the complexity of the region: multiple alleles in the region are under both positive and balancing natural selection in different populations, leading to complex long‐range haplotype structures, and many of the genes in the classical regions also show high‐sequence homology. This makes genotyping and sequencing assays technically challenging, so genotyping has remained a low‐throughput activity, in contrast to the rest of the genome, which is amenable to more scalable technologies. Over the last several years, the compilation of large reference populations with both serological and genotyping data on MHC variation has made imputation of classical alleles possible from standard SNP array data to single amino acid resolution.^48^, ^49^ We thus now have tools to interrogate this region at scale and identify the specific functional HLA alleles driving risk.

Beyond single‐marker analyses, in 2015 the IMSGC described a comprehensive dissection of allelic association in the broader MHC, based on over 48 000 samples with dense genotyping information through the region.^50^ This resulted not only in the description of multiple class II HLA‐DRB1 and HLA‐DRQ1 classical alleles imputed from SNP genotypes, but also epistatic interactions between HLA‐DQA1*01:01 and HLA‐DRB1*15:01 and between HLA‐DQB1*03:01 and HLA‐DQB1*03:02. These results raise certain functional questions, for example why the protective effect of HLA‐DQA1*01:01 only manifests in the presence of the HLA‐DRB1*15:01 risk allele.^50^, ^51^ Several variants outside the classical regions of the MHC (both class I and class II) were also shown to be independently associated, suggesting biological functions beyond antigen display underlie the MHC risk effects. However, the nature of the interaction may be more complicated than expected, with multiple different amino acid‐level alleles demonstrating consistent interaction with HLA‐DRB1*15:01, in addition to the HLA‐DQA1*01:01 allele. This suggests that the landscape of antigen presentation is very dynamic in the population and risk‐relevant phenotypes may be more complex than changes to diversity or binding strength of individual epitopes. These questions, however, will require the development of more stringent, high‐throughput experimental tools to interrogate specific HLA alleles, which we still lack.

## Identifying causal variants and pathogenic genes

As in other common, complex diseases, identifying MS risk genes has been complicated by two challenges. Firstly, identifying the causal variants in GWAS loci through fine mapping remains difficult: linkage disequilibrium means that many variants will show evidence of association to disease, but only one is likely to be the causal one. As a result of differences in minor allele frequency and population sampling, this is not necessarily the most associated variant.^22^ Fine‐mapping approaches, therefore, aim to assign posterior probabilities of causality for each variant based on some criteria. One such approach is to assess the posterior probability of causality using genotype and minor allele frequency information and then select the smallest group of variants in each locus that are likely to include the causal one at some threshold.^52^ When applied to 6356 MS cases and 9617 controls from the United Kingdom, this approach only meaningfully resolved a small subset of associations, with 8/68 loci we analysed resolving to fewer than five candidate variants, and from these, we have been able to identify a relevant candidate gene in three.^45^ This is likely a limitation in power, and larger sample sizes may help the resolution of these approaches. Alternative fine‐mapping strategies^53^, ^54^ have not yet been applied to MS data, but in other instances have performed well and are likely to prove useful in MS locus dissection.

The second challenge is that the majority of MS risk variants appear to localise to gene regulatory regions, rather than coding sequence,^55^ and specifically to enhancer elements active in stimulated immune cell subsets.^56^ MS GWAS loci are also enriched for expression quantitative trait loci (eQTL) in multiple tissues,^46^, ^57^ supporting the idea that much risk is due to changes to gene regulation. These analyses, however, aggregate information across the entire genome and do not identify individual regulatory elements relevant to disease, which remains an open question in the field. The observations of enrichment in regulatory regions engender a further conceptual challenge, as we have lacked tools to effectively predict gene targets of such regulatory elements; this is further complicated by the fact that these elements often exert their effects over considerable distances, so simple proximity‐based assignment is usually incorrect.^58^ Thus, even if fine mapping is successful in a locus, there is every chance that the relevant gene cannot be readily identified.

These discoveries have spurred efforts to integrate GWAS information with other functional genomics data to identify relevant genes. Two distinct approaches have emerged, with overlapping goals: the first is to identify genes with an eQTL driven by an MS risk variant in a locus and the second is to identify specific regulatory elements driving disease risk, and through these, the genes were affected, which must by definition be pathogenic. In attempts to overlap GWAS and eQTL data, the key issue is not just to identify eQTLs in a GWAS locus, but to identify those that appear to be driven by the same underlying genetic variant driving disease risk.^59^ This has proven a difficult challenge, as a result of linkage disequilibrium^60^. Practically, because eQTL are very common, and many variants show association to disease in a locus, it is likely that at least some variants associated with disease will also have eQTL evidence for a nearby gene.^61^ Several methods have been proposed to address this colocalisation issue,^59^, ^62^ each of which aims to compare GWAS and eQTL data to identify pleiotropic effects between them. Recently, we developed a joint likelihood approach to this problem and used it to compare MS risk associations from the IMSGC ImmunoChip study to eQTL in CD4^+^ T cells, CD14^+^ monocytes and lymphoblastoid cell lines.^63^ We found that, of 59 densely genotyped loci showing genome‐wide significance to MS risk, 56 also had an eQTL to at least one gene within 100 kb of the most associated MS variant, with most of these harbouring eQTLs to multiple genes. However, in only 14/56 loci could we find evidence that an eQTL and the MS risk effect were driven by the same underlying signal, with the remainder showing strong evidence that the genetic effects are distinct for eQTL and disease risk. This suggests that many spurious inferences will be made by simply searching for eQTL in a GWAS locus and assuming that these are causally related. For 11/14 loci, we found matches in CD4^+^ T cells, confirming the central role played by these cells in MS pathogenesis. These genes are now strong candidates for disease causality, and further work will elucidate their role in pathogenesis.

The second conceptual approach is to identify the regulatory regions driving MS risk and through these identify the relevant genes.^56^ The promise of this approach is that not only can we identify pathogenic genes, but the specific mechanisms of risk. We recently described a statistical framework to identify regions of accessible chromatin driving MS risk,^64^ using the publicly available data generated by the NIH Roadmap Epigenome Mapping Consortium^65^ (REMC). These are genomic regions of 150–400 base pairs where chromatin has been relaxed in some cell types in order to allow DNA‐binding protein interaction and is thus sensitive to cleavage by DNase I. These DNase I hypersensitive sites (DHS) usually contain transcription factor binding sites and overlap either promoter or enhancer elements.^55^ We were able to detect significant enrichment of risk alleles on open chromatin elements in 25/48 MS risk loci and that these were due to 177 DHS, of a total of > 500 000 DHS sites present in all 48 loci. We then correlated the pattern of accessibility of each of these 177 DHS sites to gene expression across REMC 56 tissues and identified 49 genes in 17/25 loci that show clear evidence of regulation by risk‐burdened DHS sites. As expected, the DHS are preferentially accessible in immune cell subsets, particularly T cells and their precursors, and the 49 genes are strongly expressed in these tissues. These genes thus form strong hypotheses about specific MS risk mechanisms in particular cells and physiological contexts.

## Pathogenic cell types and tissues

There has been long‐standing uncertainty about which specific immune cell subsets drive pathology, and what, if any, the role of the CNS is in generating risk. The vast majority of GWAS loci encode genes obviously active in the immune system,^45^, ^63^ and particularly in the lymphocyte lineage,^64^ placing beyond a doubt the nature of the disease as autoimmune. However, although the hallmark of MS pathology is the presence of oligoclonal bands in CSF, making antibody‐secreting B cells the obvious suspect, the GWAS enrichment studies all point to risk being mediated by gene regulation in CD4^+^ T cells^43^ a view reinforced by the success of α4 integrin blockade by natalizumab, which prevents T cells from crossing the blood–brain barrier and forming new lesions. However, the off‐label use of rituximab and recent approval of ocrelizumab in MS, both of which target CD20, indicate the B‐cell blockade is also effective.^66^, ^67^ Whether this is a symptom control measure rather than an attack on the root cause of disease remains to be determined.

In contrast, there has been little evidence for causal roles for CNS‐resident cells from GWAS analyses. This is in common with most other common complex autoimmune and inflammatory diseases, where gene regulation in target tissues appears to not be a major feature of GWAS loci. However, as circulating immune cells are overrepresented in most available transcriptional, epigenetic and pathway data sets, and CNS is either totally absent or represented only as gross anatomical regions, this may be due to ascertainment bias rather than underlying biology. This picture is starting to change as CNS data become more widely available. In the most recent IMSGC GWAS, 104/200 non‐MHC risk loci overlapped eQTLs active in prefrontal cortex or immune cells.^46^ These sometimes involve more than one eQTL per locus, for a total of 212 eQTLs potentially being relevant to pathogenesis. Of these, 45 are present only in prefrontal cortex and do not appear to affect gene regulation in immune cell subsets, suggesting that some effects may be restricted to CNS‐resident cells (including microglia, which are part of the hematopoietic, rather than the neural, lineage).

## Overlaps with other autoimmune diseases

As a group, the autoimmune and inflammatory diseases have proven remarkably tractable to genetic dissection in large cohorts, with several hundred risk loci now known in each disease.^68^ As these results emerged, it became obvious that many loci were associated with multiple diseases and that the genes encoded in those loci fall into distinct immune pathways.^69^, ^70^ These results suggest that perturbations to key immune processes mediate risk to multiple diseases. For example, loci encoding the core components of the IL‐23‐mediated signalling pathway mediate risk for MS, psoriasis and Crohn's disease and those involved in IL‐2‐mediated signalling with rheumatoid arthritis and type I diabetes. Notably, in some cases the allele associated with increased MS risk is associated with decreased risk to another autoimmune disease. One example is rs744166 (located in an intron of the STAT3 gene on chromosome 17): the G allele is associated with increased risk in MS^38^ and decreased risk in Crohn's disease.^71^

However, as a result of the difficulties posed by linkage disequilibrium to fine mapping and comparing across traits discussed above, claims that GWAS associations in the same region represent shared effects must be treated with caution. Nevertheless, there are clear examples of biologically plausible mechanisms: an eQTL for ankyrin D55 in CD4^+^ T cells colocalising with GWAS signals for MS, rheumatoid arthritis and Crohn's disease^63^; a specific DHS site driving risk to MS, type 1 diabetes and autoimmune thyroiditis in the MND1 locus^64^; and that T‐cell surface expression of IL‐12 receptor alpha (CD25) is associated with risk variants for both MS and type 1 diabetes.^72^ Such shared effects are interesting because they highlight more general processes of autoimmunity and therapies targeting them may show efficacy in multiple indications. However, those associations unique to MS may identify disease‐specific biology, including CNS‐relevant mechanisms.^69^

## Future directions

Genome‐wide association studies have proven remarkably successful in MS, with > 200 risk loci now identified. However, as discussed in this review, functional interpretation of these results remains a challenge, and translation to an understanding of pathobiology will remain a major target for the immediate future. The scale of the challenge is immense: from only one analysis, we have garnered 212 eQTLs that are likely to drive risk in 104 GWAS loci,^46^ and each of these will have to be followed up experimentally, possibly in multiple cell types under multiple conditions. The current model of low‐throughput, human‐operated laboratory methods simply cannot accommodate this volume of hypotheses, so the coming decade will likely see the emergence of large‐scale, automated assays in build–generate–test cycles to investigate each of these loci.

Looking beyond case–control association, several other aspects of the disease can also be dissected by genetic approaches. The largest single risk factor for MS is biological sex, with > 75% of patients being female – but the causes for this discrepancy in incidence are unknown.^73^ Approximately 95% of MS cases follow a relapsing–remitting pattern (RRMS), with approximately 50% of these converting to a secondary progressive form (SPMS) over time. The remaining 5% of all MS cases are of a more aggressive, primary progressive form (PPMS). We still do not understand the determinants of either PPMS or the risk factors for conversion from RRMS to SPMS. MS is also a remarkably heterogeneous disease, with some patients declining rapidly and others showing few or no symptoms for decades.^73^ This clinical course is unpredictable, and no tools for prognosis currently exist. Several studies have explored the genetic basis of clinical course, age of onset and severity, although no genome‐wide significant associations have been discovered.^36^, ^37^, ^43^, ^74^, ^75^, ^76^, ^77^ Whether more detailed disease parameters are more prone to error measurement, systematic differences across centres or simply not heritable remains to be determined, but a recent study showing that clinical scores can be predictive across centres suggests that lack of heritability is not the issue.^78^

Similarly, patient response to therapy is largely unpredictable; there is no evidence to date, for example, that different patients have slightly different pathologies and would thus respond to distinct modes of therapy targeting those specific pathways, although efforts to dissect this issue are underway. One of the critical barriers to large‐scale genetic mapping for these secondary characteristics is an absence of data: amassing tens of thousands of cases and controls has been daunting, but retrieving detailed disease data from medical charts written in many languages and scattered across hundreds of medical centres on several continents is the herculean task that now confronts our field. Aggregating endophenotypes such as imaging metrics, electrophysiological parameters, visual disability, biomarkers and high‐definition, computerised gait analysis, among others, may further assist in making heterogeneous clinical measurements more robust, although without integration may be difficult to interpret and will present further multiple testing challenges.

## Conflict of interest

The authors declare that no competing interests exist.
