# Genome‐wide association studies of multiple sclerosis

**DOI:** 10.1002/cti2.1038

**Published:** 2018-08-16

**Authors:** Chris Cotsapas, Mitja Mitrovic

*Clin Trans Immunol* 2018; **7**: doi: 10.1002/cti2.1038

**Correction to:** *Clin Trans Immunol* 2018; **7**: e1018;

doi: 10.1002/cti2.1018; published online 31 May 2018

The authors have realised post publication that the following reference should have been cited in the text.

Andlauer TF, Buck D, Antony G *et al*. Novel multiple sclerosis susceptibility loci implicated in epigenetic regulation. *Sci Adv* 2016; **2**: e1501678.

This article is now Reference 79 and is cited in the text on page 2 as follows:

This opened the floodgates, with several successive studies GWAS and meta‐analysis followed in rapid succession, so that by 2011, common variants in 26 genomic loci had been associated with MS risk and independently replicated, but clearly only explained a fraction of MS risk attributable to genetic factors.^33–42,79^

The authors apologise for this error.

